# Beneficial Effects of the RESMENA Dietary Pattern on Oxidative Stress in Patients Suffering from Metabolic Syndrome with Hyperglycemia Are Associated to Dietary TAC and Fruit Consumption

**DOI:** 10.3390/ijms14046903

**Published:** 2013-03-27

**Authors:** Rocio de la Iglesia, Patricia Lopez-Legarrea, Paloma Celada, Francisco J. Sánchez-Muniz, J. Alfredo Martinez, M. Angeles Zulet

**Affiliations:** 1 Department of Nutrition, Food Sciences and Physiology, University of Navarra, Pamplona 31008, Spain; E-Mails: rdelaiglesi@alumni.unav.es (R.I.); pllegarrea@alumni.unav.es (P.L.-L.); mazulet@unav.es (M.A.Z.); 2 Department of Nutrition and Bromatology, The Complutense University of Madrid, Madrid 28040, Spain; E-Mails: pcelada@ucm.es (P.C.); frasan@farm.ucm.es (F.J.S.-M.); 3 CIBERobn Physiopathology of Obesity and Nutrition, Centre of Biomedical Research Network, Madrid 29029, Spain

**Keywords:** metabolic syndrome, hyperglycemia, oxidative stress, TAC, fruit

## Abstract

Hyperglycemia and oxidative stress are conditions directly related to the metabolic syndrome (MetS), whose prevalence is increasing worldwide. This study aimed to evaluate the effectiveness of a new weight-loss dietary pattern on improving the oxidative stress status on patients suffering MetS with hyperglycemia. Seventy-nine volunteers were randomly assigned to two low-calorie diets (−30% Energy): the control diet based on the American Health Association criteria and the RESMENA diet based on a different macronutrient distribution (30% proteins, 30% lipids, 40% carbohydrates), which was characterized by an increase of the meal frequency (seven-times/day), low glycemic load, high antioxidant capacity (TAC) and high *n*-3 fatty acids content. Dietary records, anthropometrical measurements, biochemical parameters and oxidative stress biomarkers were analyzed before and after the six-month-long study. The RESMENA (Metabolic Syndrome Reduction in Navarra) diet specifically reduced the android fat mass and demonstrated more effectiveness on improving general oxidative stress through a greater decrease of oxidized LDL (oxLDL) values and protection against arylesterase depletion. Interestingly, oxLDL values were associated with dietary TAC and fruit consumption and with changes on body mass index (BMI), waist circumference, fat mass and triacilglyceride (TG) levels. In conclusion, the antioxidant properties of the RESMENA diet provide further benefits to those attributable to weight loss on patients suffering Mets with hyperglycemia.

## 1. Introduction

The prevalence of metabolic syndrome (MetS), established as the combination of central obesity and different metabolic disturbances, such as insulin resistance, hypertension and dyslipidemia, is increasing worldwide [1,2]. Among the different metabolic abnormalities encompassing MetS, insulin resistance has been considered a common manifestation of the MetS, which leads to tissue damage and health features, involving cardiovascular diseases (CVD), atherosclerosis and hypertension [3–5]. Moreover, oxidative stress has been investigated as a potential contributor to the etiology of different pathophysiological complications, including MetS and type 2 diabetes [4,6]. Therefore, many scientific efforts are under way to detect, treat and prevent MetS, focusing on lowering the risk of type 2 diabetes and oxidative stress development [7,8]. Thus, several studies have been designed and implemented to reduce these oxidative stress-related diseases based on different lifestyle modification strategies, such as giving up smoking, increasing physical activity, controlling alcohol intake, implementing healthy sleep habits, controlling anxiety and depression, losing weight and modifying unhealthy dietary patterns [7–9]. Since it has been demonstrated that central obesity is associated with increased risks of type 2 diabetes, hypertension, CVD [10,11], oxidative stress [12] and MetS manifestations in general [11], android fat mass reduction should be a main target in order to improve MetS related diseases. Concerning nutritional strategies, most of the studies have examined the effects of single dietary factors, such as the hypotriglyceridemic effect of *n*-3 fatty acids consumption [13], the protection against oxidative damage of the dietary total antioxidant capacity (TAC) [14,15], the control of blood glucose levels of low glycemic load (GL) diets [16] or the meal frequency related appetite control [17]. However, the role of a complete dietary pattern on oxidative stress and its related diseases remains unclear [18]. Thus, it was hypothesized that the combination of all these components (*n*-3 fatty acids, TAC, GL, meal frequency) may be effective when included in an integrated adequate dietary pattern. Therefore, in the present work, the effectiveness of a new dietary strategy involving different nutritional elements is studied in order to improve oxidative stress markers, as well as biochemical and body composition measurements on a population suffering MetS with hyperglycemia. The RESMENA-S (Metabolic Syndrome Reduction in Navarra-Spain) project [19,20].

## 2. Results and Discussion

### 2.1. Anthropometrical, Body Composition and Blood Pressure Parameters

After the six-month trial, both control and RESMENA dietary strategies proved to be effective on improving anthropometric, body composition and blood pressure parameters (Table 1). Both groups significantly reduced the body weight, body mass index (BMI), waist circumference, waist to hip ratio (WHR), total fat mass, lean mass, fat-free mass, systolic blood pressure (SBP) and diastolic blood pressure (DBP). However, regarding the android fat mass and related waist circumference measurement, the RESMENA diet demonstrated more benefits than the control, as volunteers of the RESMENA group presented a bigger waist circumference decrease, leading to a trend towards a marginally significance between groups (*p* = 0.060). Indeed, the RESMENA subjects were the only group that significantly reduced android fat mass values (*p* < 0.001), which resulted in significant differences between groups (*p* < 0.044). As it has been previously described, central obesity is associated with increased risks of type 2 diabetes mellitus [21], hypertension, cardiovascular diseases and MetS manifestations in general [10,11]. Moreover, only the individuals belonging to RESMENA group showed a significantly decrease in their heart rate (*p* < 0.001). Therefore, although both strategies were effective on improving general anthropometric and body composition measurements, the RESMENA diet showed additional benefits that should be taken into account in future nutritional intervention research.

Regarding physical activity, as designed, volunteers of both dietary patterns maintained their activity levels along the study, with no significant differences between groups (Table 1). Therefore, the effects on anthropometric and biochemical parameters cannot be related to changes in physical activity, but to the different dietary patterns.

### 2.2. General Biochemical Parameters

Regarding biochemical values (Table 2), both, control and RESMENA diets, proved to be effective on ameliorating the plasma biochemical profile. As it was mentioned before, insulin resistance has been postulated as a major risk condition for the MetS development [3]. Volunteers of both groups significantly reduced their insulin and Homeostasis Model Assessment Index (HOMA-IR) values, although only those under RESMENA dietary patterns ended with significantly lower glucose levels. These results agree with the review and meta-analysis carried out by Santos *et al*. [22], where it was described that caloric restriction, despite the type of diet, leads to an improvement on insulin, HOMA-IR and plasma glucose levels, but the intake of a low-carbohydrate diet demonstrated a markedly bigger effect on decreasing fasting plasma glucose levels. Since volunteers included in this study presented hyperglycemia, the fact that the RESMENA group were the only that significantly decreased the glucose values has to be highlighted and might be considered in future dietary treatments of hyperglycemic patients.

Furthermore, both dietary groups significantly reduced triglyceride (TG) values, a feature that has been associated with an amelioration of coronary heart disease risks [23]. However, concerning low density lipoprotein-cholesterol (LDL-c), unexpectedly, the two groups increased their values, results that agree with Clifton *et al*. [24], who described that in some cases, LDL-c may raise despite weight loss. However, this significant increase was not observed on apolipoprotein B (Apo B) concentrations, which has been considered a better predictor of cardiovascular disease than any other lipid measurement [25]. Moreover, according to the LDL/Apo B ratio that predicts the LDL-particle size, the values being significantly raised in both groups, it indicates an increase in LDL-particle size and a lower risk of ischemic cardiac events [26,27]. With regards to high density lipoprotein-cholesterol (HDL-c) concentrations, they rose in both groups, but this increase was statistically significant only in the control group, although apolipoprotein A-I (Apo A-I), a major protein component of HDL-c [28], did not show any changes in any of the dietary groups.

Some studies associate the rise of uric acid with gout, uric acid kidney stones, diabetes and hypertension, among other diseases [29], but it also has been proposed to have a protective role and to be able to function as an antioxidant [30]. In the present study, uric acid levels slightly raised in both groups; however, no significant differences were found, neither between day zero and 180, nor between dietary groups.

Interestingly, free fatty acids (FFA), which are known to impair aortic elastic function [31], were only significantly decreased in the RESMENA group.

Concerning renal function, low levels of estimated glomerular filtration rates (eGFRs) have been positively correlated to cardiovascular disease [32]. In the present study, the control group slightly decreased these values, whereas the RESMENA group mildly increased them, leading to a trend towards significance between groups. Although decreases in protein intake has been associated to increases of eGFRs [33], our results agree with other studies where protein intake was not associated with renal function [34,35].

Transaminases, mainly alanine aminotransferase (ALT), are markers of hepatocyte injury that have shown a correlation with insulin resistance and later development of diabetes [36]. Dietary weight loss has been associated with a depletion of this liver enzyme [37] irrespective of the type of diet [38], which agrees with the present study, where both control and RESMENA group volunteers significantly decreased their ALT levels. The control group lowered aspartate aminotransferase (AST) values, as well.

### 2.3. Oxidative Stress Biomarkers

Oxidative stress, defined as an imbalance between production and degradation of reactive oxygen species, is a potential biochemical mechanism involved in the pathogenesis of MetS and diabetes [39–41]. Therefore, the study of oxidative stress-related markers on people suffering MetS and/or diabetes is important to be approached in their treatment.

High levels of plasma malondialdehyde (MDA), a biomarker of lipid peroxidation [42], have been associated with type 2 diabetes [43]. Moreover, energy-restricted dietary strategies have demonstrated to be able to decrease MDA levels [44]. At the end of the study, both dietary treatments had reduced these biomarker levels; the control group showed statistically significant changes (*p* = 0.007), and the RESMENA group showed a trend towards significance (*p* = 0.079). When comparing both groups, no statistically significant differences were found (Table 3).

Regarding myeloperoxidase (MPO), a leucocyte-derived enzyme that catalyzes the formation of a number of reactive oxidant species and that is known to oxidize the HDL-c [45], it has been described that energy restriction diets let to depletions on its levels [46]. In the present study, both diets slightly decreased their MPO values, but no significant differences were found, neither between day zero and day 180 in each group, nor between both dietary groups (Table 3).

Arylesterase (ARE) activity, one of the three functions of the paraoxonase enzyme (PON1), is associated with HDL-c and has been shown to protect LDL-c and HDL-c against oxidation [47]. In diabetic patients, PON1 ARE activity dissociates from HDL-c [48]. Studies focusing on the effect of the diet on the ARE activity are scarce, but it has been reported that flavonoids, fish oil, nori algae and pomegranate-rich based diets are positively associated with PON1 ARE activity in diabetic patients [49–52]. In the present study, volunteers of the control diet decreased ARE:HDL-c (*p* = 0.006) and ARE:Apo A-I (*p* = 0.029) ratio values, while they remained almost unchanged in the RESMENA group. Therefore, the RESMENA diet showed a specific protection effect against ARE depletion (Table 3).

**Figure 1:**
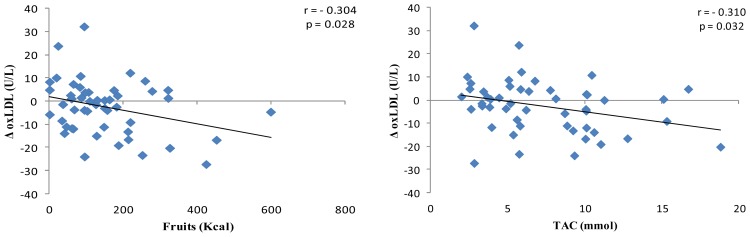
Relationship between changes on oxLDL and fruits and TAC dietary records. Abbreviations: oxLDL, oxidized low density lipoprotein; TAC, total antioxidant capacity.

**Figure 2:**
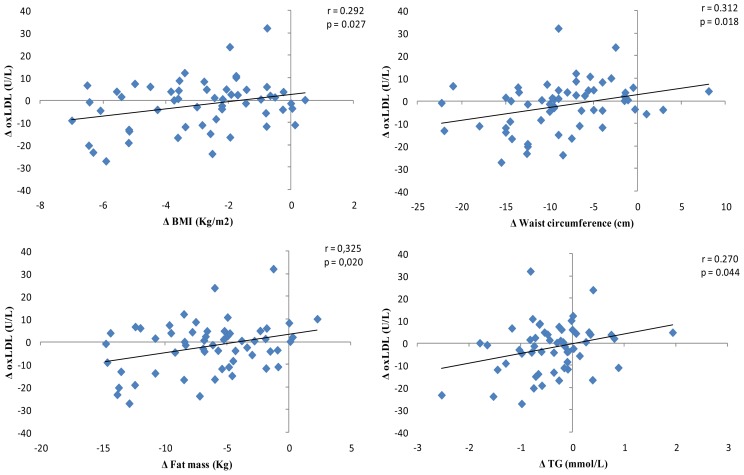
Correlations between changes on oxLDL and changes on adiposity parameters. Abbreviations: BMI, body mass index; TG, triglycerides.

Oxidation of LDL-c is considered an important cardiovascular risk factor, since it lets to foam cell formation induction, alongside propagation of atherosclerosis [53]. Moreover, oxidized-LDL (oxLDL) has been found to be a biomarker increased in type 2 diabetic patients [54]. Our results evidenced that between both dietary patterns, RESMENA is significantly more effective on reducing oxLDL (*p* = 0.025), oxLDL:LDL-c, (*p* = 0.046) and oxLDL:Apo B (*p* = 0.040) than the control diet. Moreover, the RESMENA group was the only that significantly reduced oxLDL:HDL-c values (*p* = 0.025) (Table 3). These results agree with previous studies, where an inverse relationship between high TAC dietary patterns and MetS related-oxidative stress was established [15]. Moreover, when the correlation between TAC and changes on oxLDL was studied, taking into account the entire sample, that is volunteers of both control and RESMENA groups, a significant positive relationship between oxLDL reduction and TAC values was found (Figure 1). Furthermore, the same association was observed when studying the relationship between oxLDL and consumed energy (kcal) from fruits (Figure 2). Finally, BMI, waist circumference, fat mass and TG value reductions are associated with decreases of oxLDL circulating concentration levels, taking again into account the entire sample (Figure 2). These results correlate with other studies, where a diet-induced weight loss resulted in significant reductions of oxLDL levels [46,55].

### 2.4. Dietary Records

The dietary records at the end of the study showed that the designed differences between the two dietary patterns composition were met, although no statistically significant differences were found for fiber, GL or EPA + DHA (Table 4). This outcome could be explained by the fact that the dietary records analyzed in this study were collected at the endpoint, once volunteers had completed four months of autonomy and after the six months that lasted the study. Therefore, volunteers may not complete them with the thoroughness required or might not followed the diet as strictly as at the beginning of the study. However, it was achieved that the RESMENA individuals had a higher meal frequency (*p* < 0.001), protein (*p* = 0.001) and TAC (*p* = 0.031) intake than the control group ones. Furthermore, the fruit consumption was also higher in the RESMENA group (*p* = 0.049). Moreover, both groups declared to consume the same amount of energy (Table 4), as designed. In the RESMENA group, a higher number of drop-outs than in the control group appeared, which may be a limitation of the study, although the difference was not statistically significant (*p* > 0.10).

## 3. Experimental Section

### 3.1. Subjects

**Figure 3:**
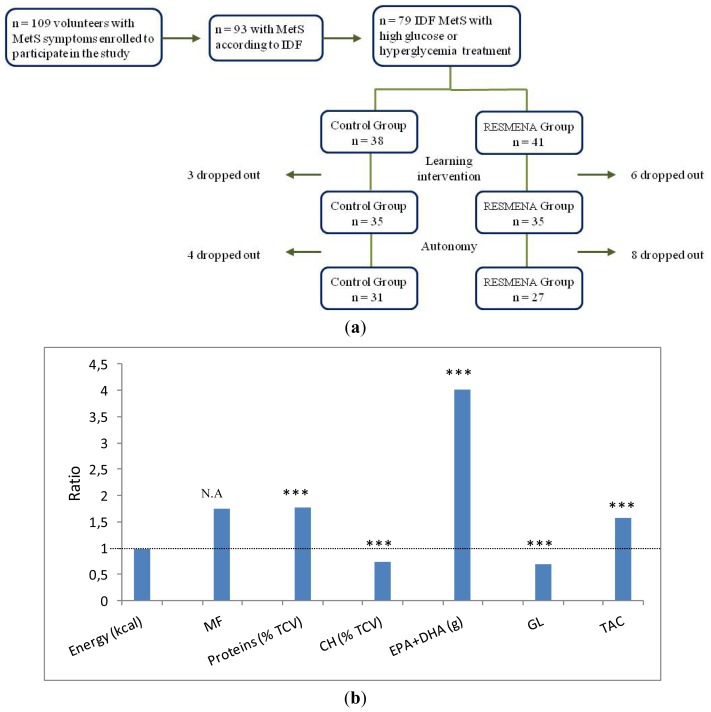
Flow diagram of participants during the study (**a**) and ratio RESMENA/control of energy and specific dietary components of the scheduled diet (**b**). Abbreviations: MetS, metabolic syndrome; IDF, International Diabetes Association; MF, meal frequency; TCV, total caloric value; CH, carbohydrates; EPA, eicosapentaenoic acid; DHA, docosahexaenoic acid; GL, glycemic load; TAC, total antioxidant capacity. Symbols: *** *p* < 0.001 differences between control and RESMENA scheduled diets; N.A, not applicable.

A subsample of 79 hyperglycemic adults diagnosed of MetS according to the IDF criteria [56] were selected from the 109 volunteers with Mets symptoms enrolled to participate in the RESMENA-S project. During the 6-month-study, 21 volunteers dropped out. Therefore, 58 individuals of the subsample completed the study and were included in the final statistical analysis (Figure 3a).

This study was conducted according to the guidelines laid down in the Declaration of Helsinki, and all procedures involving human subjects were approved by the Ethics Committee of the University of Navarra (065/2009). Written informed consent to participate in the intervention trial [20] was obtained from all subjects.

### 3.2. Study Protocol

The study was designed as a randomized, controlled trial to compare the effects of two dietary strategies (Figure 3b) on improving body composition, biochemical and oxidative stress parameters in a MetS population with hyperglycemia. Participants were randomly assigned to the control or the experimental diet (control and RESMENA groups, respectively). The study lasted a total of six months implemented in two sequential stages: an initial 8-week nutritional learning intervention period, during which the study participants received nutritional assessment every fifteen days, and a follow-up 4-month self-control period, in which they applied on their own the previously acquired nutritional habits. The CONSORT 2010 guidelines [57] were followed by taking into account the design of the present study as two-groups longitudinal intervention, except for blinding.

Participants were asked to maintain their normal physical activity during the study, which was checked by a 24-h physical activity questionnaire [58] at the beginning and at the end of the study. For assessing physical activity, all participants were asked about their occupation, sleeping hours and additional activities at work and during the rest of the day. The physical activity questionnaire included representative values expressed as multiples of Resting Energy Expenditure. Average daily physical activity level was calculated taking into account the intensity and time spent on each activity. Activities were divided in 5 categories (resting, very light, light, moderate and heavy) [58].

At baseline and at the end point of the 6-month study, trained nutritionists performed anthropometrical measurements and body composition analyses by Dual-energy X-ray Absorptiometry (DXA) following validated protocols [19]. Moreover, fasting blood samples for biochemical analyses were collected.

### 3.3. Diets

Two energy-restricted diets (−30% energy of the studied requirements) were prescribed and compared (Figure 3b). Thus, the control diet was based on the AHA guidelines [59], including 3–5 meals per day, a macronutrient distribution of 55% total caloric value (TCV) from carbohydrates, 15% proteins and 30% lipids, a healthy fatty acids (FA) profile and a cholesterol consumption lower than 300 mg/day. The RESMENA diet was characterized by a higher meal frequency, consisting of seven meals per day and by a different macronutrient distribution, 40% TCV from carbohydrates, 30% proteins and 30% lipids [19]. Furthermore, this pattern tried to reinforce the high *n*-3 polyunsaturated FA (*n*-3 PUFAs) and high natural antioxidant foods consumption and promoted low GL carbohydrates intake. It also maintained a healthy FA profile and a cholesterol content of less than 300 mg/day as the control diet.

RESMENA participants were prescribed a 7-day menu plan, while in the control group, a previously described [60] food exchange system plan was provided to volunteers. A 48-hour weighed food record was collected at the beginning and at the end of both the nutritional-learning and the autonomous periods, in order to assess the volunteer’s adherence to the prescribed nutritional patterns. The designed diets composition, as well as the different dietary records, were analyzed by the DIAL software (Alce Ingenieria, Madrid, Spain) [61]. The sum of eicosapentaenoic and docosahexaenoic fatty acid (EPA+DHA) obtained by the DIAL program [61] was used to estimate *n*-3 PUFAs consumption. TAC was calculated using the validated data, considering raw or cooked preparations [62]. Finally, the GL was obtained from the international updated website database based in the Human Nutrition Unit, School of Molecular Biosciences from the University of Sydney [63].

### 3.4. Clinical and Biochemical Assessments

Anthropometric measurements were performed in fasting conditions, as previously described [64]. Body weight was assessed to the nearest 0.1 kg by using a bioimpedance (TANITA SC-330, Tanita, Corporation, Tokyo, Japan). BMI was calculated as the body weight divided by the squared height (kg/m^2^). Waist and hip circumferences were measured with a commercial tap following validated protocols, as previously described [19]. Total body fat mass android fat mass, lean mass and fat-free mass were evaluated by DXA (Lunar iDXA™, software version 6.0, Madison, WI, USA). Measurements of SBP, DBP and heart rate were assessed using a digital monitor (Medisana, MTC, Düsseldorf, Germany) in the right arm, with the patient seated and relaxed, with an appropriate cuff for the arm size of each patient. Measurements were taken three times after a five-minute resting period, following World Health Organization (WHO) criteria [65].

Total cholesterol, HDL-c, TG, FFA, glucose, uric acid, total proteins, creatinine, ALT and AST serum concentrations were measured in an autoanalyzer Pentra C-200 (HORIBA ABX, Madrid, Spain) with specific kits. Insulin concentrations were determined by an enzyme-linked immunosorbent assay (ELISA) kit (Mercodia, Uppsala, Sweden) in a Triturus autoanalyzer (Grifols SA, Barcelona, Spain). Insulin resistance was estimated by the Homeostasis Model Assessment Index (HOMA-IR), which was calculated as stated in the following formula: HOMA-IR = [glucose (mmol/L) × insulin (μU/mL)]/22.5, as described elsewhere [66]. LDL-c levels were calculated following the Friedewald formula: LDL-c = Total cholesterol − HDL-c − TG/5 [67]. Apo A-I and Apo B were measured with specific kits (Tina-quant Apolipoprotein A-I ver.2 and Tina-quant Apolipoprotein B ver.2, Mannheim, Germany) using a Roche/Hitachi autoanalyzer (Mod.904 Modular, Tokio, Japan). Estimated glomerular filtration rates (eGFRs) were calculated from serum creatinine values using the equation CKD-EPI, which takes into account sex, age and race [68].

Plasma MDA was colorimetrically determined with a commercial kit (BIOXYTECH^®^ LPO-586™, Oxis Research™, Portland, OR, USA). Each sample (200 μL of serum) was mixed with 650 μL of *N*-methyl-2-phenylindole in acetonitrile and 150 μL of 37% (12 N) HCl. Tubes were capped, mixed and incubated at 45 °C for 60 min. Samples were centrifuged at 15,000 × *g* for 10 min, and the supernatant was read on a spectrophotometer at 586 nm (Multiskan Spectrum, Thermo Electron Corporation, Vantaa, Finland). The assay included a six-point standard curve, the measurement was performed in replicate and the mean value was computed.

Plasma ox-LDL and MPO were measured using capture ELISA assay kits from Mercodia (Uppsala, Sweden). ARE activity was measured with simulated body fluid (SBF) as buffer and phenylacetate as substrate at pH 7.34–7.4 and 37 °C, as described elsewhere [48]. Reaction rates of ARE were followed at 270 nm in thermostatically controlled 10-mm Lightpath quartz cuvettes using a Shimadzu UV-2401PC spectrophotometer (Tokio, Japan). The final reaction volume in the cuvettes was 2.0 mL, and the total time was 3 min. One unit of ARE activity is equal to 1 mol of phenylacetate hydrolyzed/(L min)

### 3.5. Statistical Analyses

Mean values and standard errors were reported for the measured variables. Differences between the beginning and the end of the complete study were analyzed by a paired *t*-test. The analysis between both groups (RESMENA *vs*. Control) was performed through an independent measures *t*-test. Correlation analyses were applied to assess the potential relationships and associations, between some components of the diet and anthropometrical and biochemical parameters variation. For drop-out analysis, the χ^2^ test was applied. The SPSS 15.1 software for Windows (SPSS Inc., Chicago, USA) was used for all statistical analyses. Values of *p* < 0.05 were considered as statistically significant.

## 4. Conclusions

Both energy-restricted dietary patterns, AHA guidelines-based diet and the RESMENA diet were successful on improving anthropometrical measurements, body composition, blood pressure levels and biochemical markers on patients suffering MetS with hyperglycemia. However, the RESMENA diet showed greater benefits regarding android fat mass reduction and improvement of the general oxidative stress status, specifically oxLDL related markers. Interestingly, dietary TAC and fruit consumption were apparently the nutritional components that potentially contributed most to the oxLDL depletion. Moreover, the decrease on BMI, waist circumference, fat mass and TG levels were also directly associated with the oxLDL decrease levels. For all of this, the prescription of the RESMENA diet is a good antioxidant dietary treatment for people suffering MetS with hyperglycemia to further improve the benefits associated to weight loss.

## Figures and Tables

**Table 1:** Changes in anthropometric parameters, body composition, blood pressure and activity level in both experimental groups (control and Metabolic Syndrome Reduction in Navarra (RESMENA)).

	Control		RESMENA		*P*^†^ Difference
	
	Day 0	Day 180	Day 0	Day 180	
**Weight (kg)**	103.1 ± 2.9	95.35 ± 2.9 ***	106.0 ± 3.2	96.7 ± 3.0 ***	0.281
**BMI (kg/m^2^)**	36.4 ± 0.7	33.7 ± 0.8 ***	37.41 ± 0.8	34.12 ± 0.8 ***	0.206
**Waist circumference (cm)**	114.6 ± 2.0	107.4 ± 2.0 ***	117.2 ± 2.1	107.1 ± 2.0 ***	0.060
**WHR**	1.00 ± 0.02	0.97 ± 0.02 ***	0.99 ± 0.02	0.95 ± 0.02 ***	0.098
**Total fat Mass (kg)**	42.3 ± 1.5	36.4 ± 1.6 ***	45.4 ± 1.9	37.9 ± 1.8 ***	0.139
**Android Fat Mass (kg)**	4.7 ± 0.2	4.3 ± 0.3	5.3 ± 0.2	4.0 ± 0.2 ***	0.044
**Lean mass (kg)**	58.0 ± 2.2	55.6 ± 2.1 ***	57.1 ± 2.1	55.5 ± 2.0 **	0.197
**Fat-free mass (kg)**	60.9 ± 2.3	58.6 ± 2.2 ***	60.0 ± 2.1	58.4 ± 2.1 **	0.220
**SBP (mmHg)**	152.9 ± 3.3	138.7 ± 2.2 **	154.2 ± 4.4	137.1 ± 3.1 **	0.637
**DBP (mmHg)**	86.3 ± 1.6	79.2 ± 1.8 **	85.8 ± 1.8	79.5 ± 2.0 ^*^	0.766
**Heart rate (bpm)**	75 ± 3	72 ± 3	82.3 ± 2.6	72.1 ± 2.5 ***	0.587
**Activity level**^1^	1.59 ± 0.04	1.54 ± 0.04	1.54 ± 0.03	1.55 ± 0.03	0.191

Abbreviations: BMI, body mass index; WHR, waist to hip ratio; SBP, systolic blood pressure; DBP, diastolic blood pressure; Symbols:

** *p* < 0.005;

*** *p* < 0.001 (comparison between day 0 and day 180 in each group);

*P*^†^, comparison between dietary group differences.

1 Average daily exercise calculated by twenty forth physical activity questionnaire.

**Table 2:** Changes in biochemical parameters in both experimental groups (control and RESMENA).

	Control		RESMENA		*P*^†^ Difference
	
	Day 0	Day 180	Day 0	Day 180	
**Total Cholesterol (mmol/L)**	5.56 ± 0.19	5.66 ± 0.19	5.44 ± 0.21	5.44 ± 0.20	0.397
**HDL-c (mmol/L)**	1.14 ± 0.05	1.28 ± 0.06 ***	1.11 ± 0.04	1.15 ± 0.04	0.057
**LDL-c (mmol/L)**	3.47 ± 0.18	4.38 ± 0.17 ***	3.34 ± 0.17	4.29 ± 0.19 ***	0.884
**LDL-c/ApoB**	1.43 ± 0.04	1.91 ± 0.04 ***	1.50 ± 0.11	1.92 ± 0.03 **	0.593
**TG (mmol/L)**	2.06 ± 0.21	1.67 ± 0.21 *	2.17 ± 0.21	1.72 ± 0.20 **	0.574
**Apo A-I (mg/dL)**	134.3 ± 4.3	139.2 ± 4.1	126.3 ± 3.5	131.2 ± 4.3	0.978
**Apo B (mg/dL)**	93.4 ± 3.7	88.7 ± 3.4	90.3 ± 4.6	86.9 ± 4.1	0.737
**FFA (mmol/L)**	0.55 ± 0.04	0.48 ± 0.04	0.60 ± 0.18	0.50 ± 0.23 *	0.349
**Glucose (mmol/L)**	7.14 ± 0.36	6.68 ± 0.28	7.59 ± 0.43	6.49 ± 0.35 **	0.118
**Insulin (μU/mL)**	15.22 ± 1.56	10.01 ± 1.54 ***	15.36 ± 1.53	9.41 ± 1.21 ***	0.685
**HOMA-IR**	4.92 ± 0.55	3.25 ± 0.61 **	5.24 ± 0.56	2.80 ± 0.37 ***	0.475
**Uric Acid (mg/dL)**	6.08 ± 0.21	6.29 ± 0.22	6.19 ± 0.28	6.23 ± 0.22	0.310
**Total Proteins (mg/dL)**	73.01 ± 0.94	76.30 ± 1.19 ***	71.48 ± 0.79	73.51 ± 0.97 *	0.186
**eGFRs (mL/min/1.73 m^2^)**	83.97 ± 2.92	79.85 ± 2.60	79.07 ± 2.72	81.46 ± 3.08	0.080
**ALT (U/L)**	41.59 ± 4.29	27.16 ± 1.56 **	28.90 ± 2.13	22.54 ± 1.60 **	0.172
**AST (U/L)**	27.73 ± 2.26	22.86 ± 1.15*	22.68 ± 1.08	20.38 ± 1.00	0.685

Abbreviations: HDL-c, high density lipoprotein cholesterol; LDL-c, low density lipoprotein cholesterol; TG, triacilglycerides; Apo A-I, apolipoprotein A-I; Apo B, apolipoprotein B; FFA, free fatty acids; HOMA-IR, homeostasis model assessment of insulin resistance; eGFRs, estimated glomerular filtration rates; ALT, alanine aminotranferase; AST, aspartate aminotransferase. Symbols:

* *p* < 0.05;

** *p* < 0.005;

*** *p* < 0.001 (comparison between day zero and day 180 in each group);

*P*^†^, comparison between dietary group differences.

**Table 3:** Changes in oxidative stress parameters in both experimental groups (control and RESMENA).

	Control		RESMENA		*P*^†^ Difference
	
	Day 0	Day 180	Day 0	Day 180	
**MDA (μM)**	0.86 ± 0.07	0.75 ± 0.07 *	0.83 ± 0.07	0.76 ± 0.05	0.449
**MPO (μg/L)**	71.69 ± 7.36	65.39 ± 7.65	69.53 ± 8.39	66.48 ± 7.42	0.723
**ARE (U/L)**	458 ± 44	442 ± 43	370 ± 31	361 ± 28	0.778
**ARE:HDL-c (U/mmol)**	413.6 ± 0.1	366.8 ± 0.1 *	343.8 ± 0.1	327.1 ± 0.1	0.227
**ARE:Apo A-I (U/mg)**	0.347 ± 0.030	0.319 ± 0.027 *	0.295 ± 0.024	0.281 ± 0.022	0.424
**oxLDL (U/L)**	35.36 ± 1.80	36.39 ± 2.60	46.53 ± 4.46	41.03 ± 3.22 *	0.025
**oxLDL:LDL-c (U/mmol)**	10.34 ± 0.52	8.25 ± 0.62 **	14.88 ± 1.80	9.52 ± 0.58 **	0.046
**oxLDL:HDL-c (U/mmol)**	30.89 ± 1.52	28.46 ± 1.76	42.78 ± 4.19	4.19 ± 2.64 *	0.186
**oxLDL:Apo B (U/mg)**	0.038 ± 0.002	0.043 ± 0.004	0.051 ± 0.004	0.048 ± 0.003	0.040

Abbreviations: MDA, malondialdehyde; MPO, myeloperoxidase; ARE, arylesterase; HDL-c, high density lipoprotein-cholesterol; ApoA1, apolipoprotein A1; oxLDL, oxidized low density lipoprotein; LDL-c, low density lipoprotein-cholesterol; ApoB, apolipoprotein B. Symbols:

* *p* < 0.05;

** *p* < 0.005;

*** *p* < 0.001 (comparison between day zero and day 180 in each group);

*P*^†^, comparison between dietary group differences.

**Table 4:** Comparison of control and RESMENA dietary records at the endpoint.

	Control	RESMENA	*p*
Energy (kcal/day)	1513 ± 54	1569 ± 77	0.542
Meal Frequency (meals/day)	4.3 ± 0.2	5.8 ± 0.2	<0.001
Proteins (% TCV/day)	16.9 ± 0.4	20.4 ± 0.9	0.001
Lipids (% TCV/day)	40.8 ± 1.5	37.7 ± 1.0	0.108
CHO (% TCV/day)	37.1 ± 1.5	36.9 ± 1.1	0.940
Fiber (% TCHO/day)	11.4 ± 0.8	12.0 ± 0.6	0.573
GL (U/day)	73.4 ± 5.9	70.0 ± 5.5	0.682
EPA+DHA (g/day)	0.30 ± 0.08	0.39 ± 0.17	0.617
TAC (mmol/day)	6.1 ± 0.6	8.5 ± 0.9	0.031
Fruits (kcal/day)	117 ± 21	185 ± 27	0.049

Abbreviations: TCV, total caloric value; CHO, carbohydrates (without fiber); TCHO, total carbohydrates (included fiber); GL, glycemic load; EPA, eicosapentaenoic acid; DHA, docosahexaenoic acid; TAC, total antioxidant capacity.
